# Franz Cell Diffusion Testing and Quantitative Confocal Raman Spectroscopy: In Vitro-In Vivo Correlation

**DOI:** 10.3390/pharmaceutics12090887

**Published:** 2020-09-18

**Authors:** Fotis Iliopoulos, Peter J. Caspers, Gerwin J. Puppels, Majella E. Lane

**Affiliations:** 1 School of Pharmacy, University College London, 29-39 Brunswick Square, London WC1N 1AX, UK; m.lane@ucl.ac.uk; 2 RiverD International B.V., Marconistraat 16, 3029 AK Rotterdam, The Netherlands; pcaspers@riverd.com (P.J.C.); gpuppels@riverd.com (G.J.P.); 3 Center for Optical Diagnostics and Therapy, Department of Dermatology, Erasmus MC, University Medical Center, 3015 CN Rotterdam, The Netherlands

**Keywords:** Confocal Raman Spectroscopy, bioequivalence, niacinamide, in vitro-in vivo correlation, skin

## Abstract

Previously, we reported the use of Confocal Raman Spectroscopy (CRS) to investigate the topical delivery of actives and excipients. We have also correlated the results from CRS with findings from in vitro diffusion studies in human skin. However, until now CRS has only been used as a semi-quantitative method of determining the skin uptake of molecules, with results expressed as arbitrary units of signal intensity. Clearly, this posed challenges for using CRS to determine skin delivery and to assess the drug bioavailability and bioequivalence of topical formulations. In the present work, the permeation of niacinamide (NIA) from various formulations in human skin was studied in vitro using conventional Franz cells and in vivo using a quantitative CRS method under finite dose conditions. The selection of NIA was based on its wide use in pharmaceutical and personal care formulations for many years. This is the first fully quantitative study to compare these methods. The vehicles investigated were neat Transcutol^®^ P (TC); binary combinations of propylene glycol (PG) with propylene glycol monolaurate (PGML); and ternary mixtures of PG, PGML, and isopropyl myristate (IPM). These solvents were selected to encompass a range of physicochemical properties. NIA permeation was evident from all formulations in vitro and in vivo. The vehicles PG:PGML and PG:PGML:IPM delivered comparable amounts across the skin in vitro at 24 h (100.3–106.7 µg/cm^2^, *p* > 0.05) that were significantly higher compared with those of TC (1.3 µg/cm^2^, *p* < 0.05). An excellent in vitro in vivo correlation (R^2^ = 0.98) was found following the linear regression of the cumulative amounts of NIA permeated in vitro and the amounts of NIA at 2 μm in the skin measured with CRS. A very good correlation between the cumulative permeation of NIA in vitro and the total amount of NIA that penetrated the stratum corneum (SC) per unit of surface area (μg/cm^2^) in vivo was also observed, with a Pearson correlation coefficient (R^2^) of 0.94. The findings support the use of CRS for the quantitative measurement of actives delivered to the skin in vivo. Future studies will focus on exploring the reproducibility and reliability of the method by investigating the delivery of different actives from a wider range of vehicles. Additionally, quantitative CRS will be evaluated further as a method for assessing the bioequivalence of topical formulations.

## 1. Introduction

Confocal Raman Spectroscopy (CRS) is an optical method that combines the principles of confocal microscopy and Raman spectroscopy. The application of CRS in skin research was introduced by Caspers et al. [1], and enabled the determination of the molecular composition of the skin as a function of depth, both in vitro and in vivo. CRS is a non-invasive technique and has been used for several applications, including the collection of depth profiles of substances in the skin, such as water [2,3] and endogenous amino-acids or ceramides [4,5,6] in real-time. CRS has also been used to estimate the stratum corneum (SC) thickness [7,8,9,10], and to investigate the in vivo penetration of topically applied substances [11,12,13]. 

The use of accurate and reproducible methods for the assessment of the percutaneous absorption of drugs is essential for the quality and safety evaluation of pharmaceutical formulations. These methods can, where validated appropriately, also be used for the determination of the bioequivalence of topical products or drug bioavailability. To date, however, only a few experimental approaches have been established to have such reliability. Among them, the in vitro permeation test (IVPT) in human skin is considered a “gold-standard” method for studying percutaneous absorption, with good correlations with human in vivo data [14,15,16,17]. In addition to the use of IVPT to screen and optimize topical formulations, the method has also been proposed for the bioequivalence testing of topical formulations. Franz et al. [18] investigated the percutaneous absorption of various active pharmaceutical ingredients (tretinoin, alclometasone dipropionate, halobetasol propionate, and mometasone furoate) from seven generic topical formulations (two tretinoin gels and five glucocorticoid creams and ointments), using finite dose (5 μL) diffusion studies in excised human skin in vitro. The total drug absorption was compared with the permeation values from the corresponding Reference Listed Drug products (RLDs). An RLD is an approved product to which new generic pharmaceutical drug products are compared to confirm that they are bioequivalent and interchangeable when administered under similar conditions [19]. In six of the seven cases, the in vitro test to reference ratio for total absorption was close to one, indicating equivalence between the formulations. These data were subsequently compared with in vivo findings following clinical trials or vasoconstrictor assays, and were found to be in agreement with the clinical data. The potential of IVPT to correlate with in vivo results was further examined by Lehman et al. [20]. In a meta-analysis, these researchers collated data for the human percutaneous absorption of permeants measured under in vitro and in vivo conditions. A positive correlation of the in vitro permeation data and percentage of applied dose delivered in vivo was reported for 30 compounds from 92 data sets, with an average in vitro-in vivo (IVIV) correlation ratio of 1.6. However, the data obtained from the in vitro experiments involved different experimental parameters compared with those utilized in vivo i.e., different application skin sites or alternative vehicles. To minimise the variations in skin absorption because of these differences, the researchers identified 11 data sets from studies that were performed under similar protocols (harmonised study designs). Subsequently, an excellent average IVIV ratio of 0.96 was found for these studies, demonstrating the ability of the IVPT model to predict the in vivo performance of formulations in humans.

There is an urgent need for new approaches to determine the bioavailability of topically applied drugs, as highlighted in an expert review summarising the proceedings of a workshop for establishing the therapeutic bioequivalence of dermatologic topical products [21]. Previously, we have shown that the skin uptake of actives measured with CRS correlated well with human skin in vitro permeation data. A robust IVIV correlation between CRS with a method that has been well-established for skin absorption studies is essential for the validation of CRS for the quantitative measurement of active delivery to skin and bioequivalence assessment. Mohammed et al. [22] investigated the percutaneous absorption of niacinamide (NIA) in human skin in vitro and in vivo. The in vitro studies were conducted using conventional Franz cells, while the in vivo experiments were carried out by CRS following a 30 min application of formulations. The experiments were performed under infinite dose conditions, thus enabling the calculation of the steady-state flux of NIA for the various vehicles in vitro. The in vitro flux of NIA was linearly proportional to the signal intensity of the compound at a depth of 4 μm in the SC in vivo after a 30 min application. Values for the correlation coefficients (R^2^) were found to be 0.96 and 0.91 for NIA flux values greater or lower than 10 μg/cm^2^/h, respectively. In a later study, similar experiments were conducted by Mateus et al. [23] to investigate the percutaneous absorption of salicylic acid in vitro and in vivo, although these researchers used finite doses (10 mg/cm^2^) of formulations. The in vitro studies were conducted in porcine ear skin, an acceptable surrogate for human skin. The in vivo studies involved CRS measurements in three human subjects following a 30 min application of formulations. A linear correlation (R^2^ = 0.98) was reported between the cumulative permeation of salicylic acid in vitro and the CRS signal of the compound at a skin depth of 2 μm in vivo. 

Although these studies from our group have demonstrated positive IVIV correlations, the CRS data were expressed as arbitrary units of intensity, thus providing relative measurements rather than absolute amounts of drug or active in the skin. The lack of quantifiable concentration profiles of actives has thus limited the use of CRS to date in studying the delivery of molecules to the skin in vivo [17]. Recently, Caspers et al. [24] proposed a method to quantify the permeation of compounds following CRS analysis. These researchers demonstrated a mathematical procedure for the calculation of the mass ratio of the permeant (mg) per protein (g) based on the corresponding Raman signal intensity ratio. Calculations of the permeant concentration inside the skin (mg/cm^3^) as well as the cumulative permeation per unit skin area (μg/cm^2^) were reported. The in vivo permeation data of a personal care ingredient, trans-retinol, as reported in 2007 by Pudney et al. [12], were re-analyzed using this new quantitative approach. Although this report was encouraging, to date there are no published studies that have compared or correlated the quantitative CRS with other well-established and validated methods.

The aims of the present work were therefore (i) to investigate the human skin permeation of niacinamide (NIA) using both Franz cell diffusion in vitro studies and quantitative CRS in vivo studies and (ii) to compare the in vitro and in vivo findings. In this proof-of-concept study, in vitro studies were conducted on skin from one donor, and in vivo spectroscopic data were collected from one human subject in order to minimize biovariability, as also described in previous work [22]. NIA was selected as a model active because it has been used in a range of pharmaceutical formulations, [25] and the ability of NIA to permeate the skin has been demonstrated in a number of studies [26,27]. 

## 2. Materials and Methods 

### 2.1. Materials

Niacinamide (NIA), 1,2 propanediol (PG), and isopropyl myristate (IPM) were supplied by Sigma-Aldrich (Dorset, UK). Lauroglycol™ 90 or propylene glycol monolaurate Type II (PGML) and Transcutol^®^ P or ethoxydiglycol (TC) were kind donations from Gattefossé (Saint-Priest, France). Phosphate buffered saline (PBS) tablets were purchased from Oxoid Limited (Cheshire, UK). HPLC-grade water and HPLC-grade methanol were obtained from Fisher Scientific (Leicestershire, UK). Full-thickness abdominal human skin was obtained from Ethical Tissue (University of Bradford, UK) following plastic surgery from a single donor with institutional ethical approval and informed consent (Research Ethics Committee reference 07/H1306/98). The epidermis was separated by the heat separation method according to procedures reported previously [28]. Briefly, the adipose tissue of the full-thickness human skin was removed using a scalpel and the skin was immersed in water at 60 °C for 45 s using blunt forceps. The tissue was removed from the water and pinned on a corkboard. Subsequently, the epidermis was carefully peeled away from the dermis in a single sheet. The epidermis was stored at −20 °C until required.

### 2.2. Methods

#### 2.2.1. HPLC Analysis

The HPLC method for the analysis of NIA has been reported and validated in earlier work [29]. Briefly, analysis was performed using a Luna^®^ reverse phase column, 250 × 4.60 mm, 5 μm, Phenyl-Hexyl (Phenomenex, Macclesfield, UK), and a universal HPLC guard column (Phenomenex, Macclesfield, UK) packed with a SecurityGuard™ cartridge (Phenomenex, Macclesfield, UK). The mobile phase consisted of methanol:water (20:80, *v*/*v*) and the flow rate was 1 mL/min. The UV detector was set to 263 nm and the column temperature to 30 °C. The injection volume was 10 μL. Calibration curves for NIA within the concentration range of 0.5 to 200 µg/mL were constructed (r^2^ ≥ 0.99), with the limit of quantification (LOQ) being 0.47 µg/mL.

#### 2.2.2. In Vitro Human Skin Permeation Studies 

Finite dose human skin permeation studies were conducted using vertical glass Franz-diffusion cells (Soham Scientific, Ely, Cambridgeshire, UK) according to the Organisation for Economic Co-operation and Development (OECD) guidelines [30,31]. The diffusion area of the donor chamber was ~1 cm^2^, accurately measured for each cell using an electronic digital micrometer (Fisher Scientific, Leicestershire, UK). Approximately 2 mL of freshly prepared phosphate buffered saline (PBS), pH = 7.3 ± 0.2, was used as the receptor solution. The experiments were conducted in a temperature-controlled water bath to ensure a skin surface temperature of 32 ± 1 °C. A dose of 5 μL of the formulations was applied to the donor compartment using a micropipette. A volume of 200 μL of receptor fluid was collected at different time intervals up to 24 h, and an equal volume of fresh temperature-equilibrated PBS solution was added to the receptor compartment. All the samples were analyzed using the validated HPLC method for NIA. Solutions of NIA (3% *w*/*w*) were prepared in three formulations: TC, PG:PGML (0.75:0.25), and PG:PGML:IPM (0.65:0.30:0.05). This concentration of NIA is commonly used in topical preparations [26], and these formulations are known to promote NIA permeation through skin [29,32]. The selection of TC as a single solvent was based on its reported efficacy in promoting NIA skin delivery compared with other neat solvents [29]. Prior to the permeation experiments, a section of frozen skin was cut and placed in a basin of distilled water. The skin was mounted on a piece of filter paper (Fisherbrand™ Grade 601, Fisher Scientific, Leicestershire, UK) and was subsequently cut into pieces of appropriate size before assembly in the Franz cells. The integrity of human skin was examined by measuring the impedance of the skin [33]. The number of replicate experiments was 4 ≥ *n* ≥ 3. 

At the end of the permeation experiments, mass balance studies were conducted to measure the amounts of NIA on the surface and inside the skin, according to procedures described previously [29,32]. Briefly, the skin surface was washed with three consecutive 1 mL volumes of water:methanol (50:50, *v*/*v*) and was subsequently dried with a cotton swab. The Franz cells were then disassembled and the skin membranes were extracted with 1 mL of water:methanol (50:50, *v*/*v*) by incubation in an orbital shaker (Orbital Mini shaker, VWR International Limited, Leicestershire, UK) at 32 °C overnight. All the samples were centrifuged at 13,000 rpm at 32 °C for 20 min, and aliquots from the supernatant solutions were collected and analyzed using the HPLC analytical method.

#### 2.2.3. Confocal Raman Spectroscopy

Raman measurements were obtained in vivo with a gen2-SCA confocal Raman spectrometer (RiverD International B.V., Rotterdam, The Netherlands). The research protocol was approved by the UCL Research Ethics Committee (Reference number: 16883/001, 7 January 2020). The instrument was equipped with two fiber-coupled diode pumped lasers of two different wavelengths: 671 and 785 nm. These wavelengths were used to record spectra in the high wavenumber (HWN) (2500–4000 cm^−1^) and the fingerprint (FP) (400–1800 cm^−1^) region, respectively. On the day of the experiment, a National Institute of Standards and Technology (NIST) glass calibration standard was positioned over the microscope objective, and the instrument was calibrated as described elsewhere [24]. The Raman spectra of human skin were taken in both the FP and the HWN region. The FP scans were carried out with a 5 s exposure time and 2 μm steps to a final depth of 28 μm. For the HWN measurements, 0.5 s exposure times and 2 μm steps were used to a final depth of 40 μm. For the quantification of NIA, the SkinTools 3 analysis software (RiverD International B.V., version 3.3.20200720, Rotterdam, The Netherlands) was used according to procedures described by Caspers et al. [24]. 

A finite dose, 5 μL/cm^2^, of formulation was applied evenly over the marked area with a pipette and tip, without rubbing. The formulations tested were 3% (*w*/*w*) NIA in TC, PG:PGML (0.75:0.25), and PG:PGML:IPM (0.65:0.30:0.05), as for the in vitro experiments. An application site measuring 3.8 cm^2^ was delineated on the volar forearm of one single volunteer (male, Caucasian, 29). The formulations were left on the skin for 60 min, and subsequently were cleared with a cotton bud before analysis. The distance between the selected points of the applied area for analysis was greater than 25 μm. On another application site, a series of scans were taken with the 785 nm finger print region laser, and served as baseline measurements. 

#### 2.2.4. Data Analysis 

Data were processed with SkinTools 3 (RiverD International B.V., Rotterdam, The Netherlands) and plotted using the GraphPad Prism Statistics software (GraphPad Software LLC, version 8.3.0, San Diego, CA, USA, 2019). The results obtained for each formulation were averaged to yield a single concentration profile. Normality was assessed using the Kolmogorov–Smirnov statistical test. For parametric data, statistical evaluation was performed by a one-way analysis of variance (ANOVA), and multiple comparisons between groups by a post hoc Tukey test. For non-parametric data, the Kruskal–Wallis test was performed. A probability of *p*  <  0.05 was considered statistically significant. The results are presented as mean ± standard deviation (SD) unless otherwise stated. For the CRS data, the baseline fit contributions of NIA were subtracted from the measurements, and the results are shown as mean ± standard error of the mean (SEM). The IVIV correlations were plotted as mean ± SEM and were calculated using Pearson’s correlation coefficient (R^2^).

## 3. Results and Discussion

### 3.1. In Vitro Human Skin Permeation Studies

**Figure 1:**
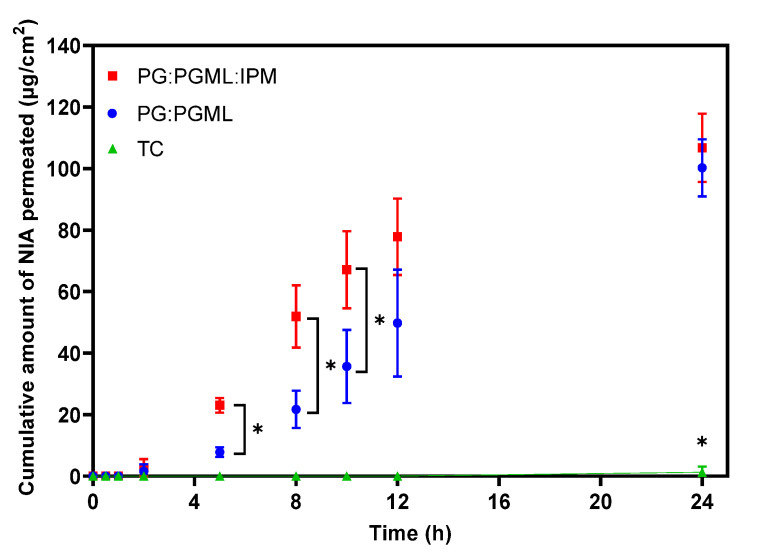
Cumulative permeation of NIA over time for various solvent systems in the human epidermis (4 ≥ *n* ≥ 3; mean ± SD, one-way ANOVA, * *p* < 0.05).

Finite dose permeation studies of NIA were performed for 24 h, and the results are shown in Figure 1.

The binary and ternary systems, PG:PGML and PG:PGML:IPM, delivered comparable amounts of NIA across the skin over a period of 24 h i.e., 100.3 ± 10.8 µg/cm^2^ and 106.7 ± 12.9 µg/cm^2^, respectively (*p* > 0.05). Although both vehicles delivered similar amounts of NIA at 24 h, the formulation comprising PG:PGML:IPM outperformed the one comprising PG:PGML at the earlier time points i.e., at 5, 8, and 10 h (*p* < 0.05). With regard to the neat TC, this vehicle delivered 1.3 ± 2.3 µg/cm^2^ of NIA at 24 h. The NIA cumulative permeation from TC was significantly lower than all the other formulations (*p* < 0.05). As regards the more complex vehicles, their effects on NIA permeation have not been previously investigated. However, both these vehicles were reported to be effective in promoting the permeation of the hydrophilic molecule 3-*O*-ethyl-l-ascorbic acid in porcine skin under finite dose conditions [34]. The penetration enhancement observed was attributed to the synergistic effects of PG with the fatty acid esters PGML and IPM. In an earlier study, Mohammed et al. [22] examined the effects of a vehicle comprising PG:PGML (50:50) on NIA permeation in human skin in vitro and in vivo. These researchers also reported the synergy of these solvents, resulting in greater NIA flux (96.1 μg/cm^2^/h) compared with neat PG (0.2 μg/cm^2^/h), although the statistical significance of this enhancement was not reported. The impact of IPM on the skin barrier has been examined by a number of researchers. It is generally proposed that the solvent may intercalate with the SC lipids and exert a disordering effect, thereby promoting the permeation of certain compounds across the barrier [35,36,37,38]. 

**Figure 2:**
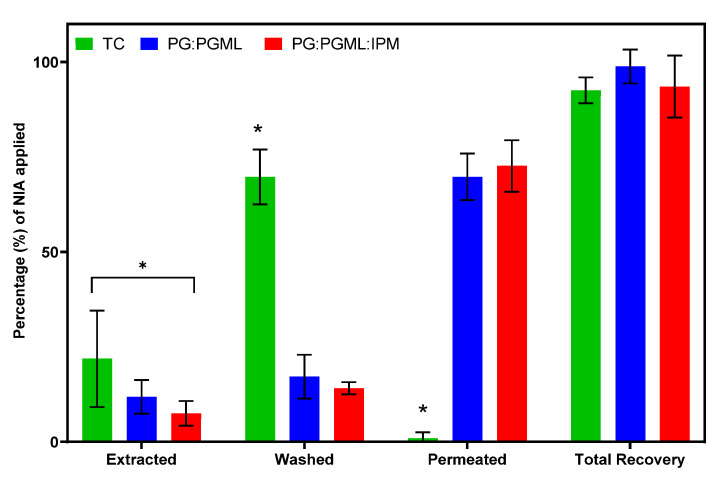
Percentage (%) recovery values of NIA for various solvent systems following mass balance studies in human epidermis (4 ≥ *n* ≥ 3; mean ± SD, one-way ANOVA, * *p* < 0.05).

The results for the mass balance studies for NIA formulations are shown in Figure 2.

Figure 2 shows that the PG:PGML:IPM delivered 72.6% of the applied NIA across the skin at 24 h, and this value was similar to the percentage permeation of NIA from PG:PGML (69.8%, *p* > 0.05). Both vehicles were more effective than TC, which delivered 0.9% of the applied NIA (*p* < 0.05). For TC, most of the applied NIA remained on the surface of the skin (69.7%). This value was significantly higher compared with those of the other vehicles, which ranged from 14.1% to 17.2%, (*p* < 0.05). The percentage of applied NIA retained inside the epidermis for the neat solvent TC (21.9%) was significantly higher than that from the PG:PGML:IPM (7.5%, *p* < 0.05), but similar to that of PG:PGML (11.9%, *p* > 0.05). The high skin retention of NIA from the solvent TC was also reported in a previous study in the human epidermis in the literature, where 32.9% of the applied dose was extracted following the application of 5 μL of NIA 5% (*w*/*v*) in vitro [39]. Finally, the total recoveries of NIA were not statistically different among the formulations and ranged from 92.5% to 98.8% (*p* > 0.05). These values were within the acceptable range of 80–120% established by the OECD guidelines [30], and indicate the stability of NIA during the permeation experiment.

### 3.2. Confocal Raman Spectroscopy

The SC thickness was estimated based on the water concentration profiles obtained in the HWN region according to procedures described in previous studies in the literature [7,8,40]. The water concentration profiles are calculated by the protein/water ratios following the integration of the corresponding peaks of the spectra obtained at sequential depth increments. Here, the SC thickness of the volunteer was 18.2 μm, calculated from an average of eight different readings.

**Figure 3:**
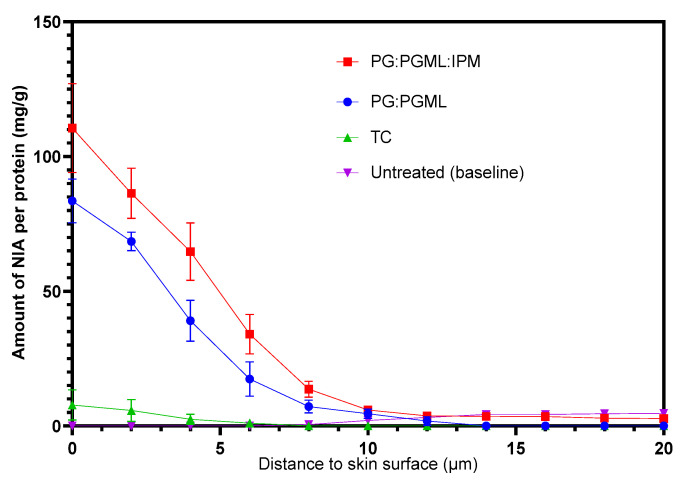
Depth profiles of NIA (mg/g) across the human volar forearm skin as a function of distance to the skin surface following a 60 min application of formulations in vivo (*n* = 8; mean ± SEM). The untreated skin served as a baseline and has been subtracted from the other profiles.

The CRS signal was quantified and the concentration profiles of NIA were expressed as the amount of NIA permeated (mg) per protein (g). The NIA depth profiles are shown in Figure 3.

**Figure 4:**
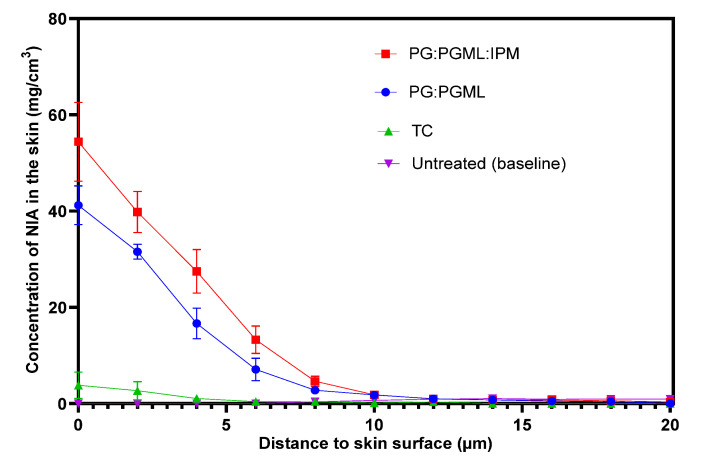
Concentration profiles of NIA (mg/cm^3^) across the human volar forearm skin as a function of distance to the skin surface following a 60 min application of formulations in vivo (*n* = 8; mean ± SEM). The untreated skin served as a baseline and has been subtracted from the other profiles.

Overall, the amounts of NIA permeated from the solvent systems PG:PGML and PG:PGML:IPM were found to show no statistical differences at each depth interval (*p* > 0.05). Quantitative analysis also enabled the determination of the NIA concentration profiles in the skin, expressed as mg/cm^3^. The results are shown in Figure 4.

**Figure 5:**
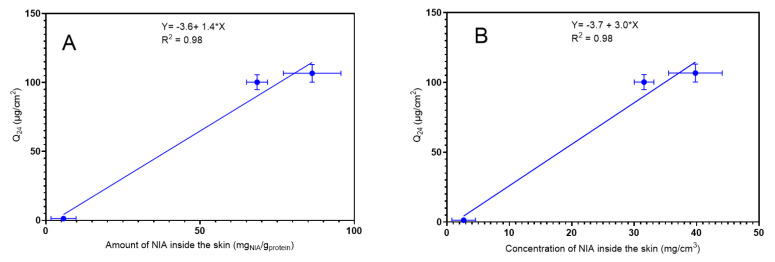
(**A**) Correlation of the in vitro cumulative amount of NIA permeated through human skin (4 ≥ *n* ≥ 3; mean ± SEM) and the in vivo amount of NIA inside the skin at 2 μm after the 60 min application of formulations (*n* = 8; mean ± SEM). (**B**) Correlation of the in vitro cumulative amount of NIA permeated through human skin (4 ≥ *n* ≥ 3; mean ± SEM) and the in vivo concentration of NIA inside the skin (mg/cm^3^) at 2 μm after the 60 min application of formulations (*n* = 8; mean ± SEM).

The correlation between the in vitro permeation studies using human skin and the in vivo topical delivery of NIA was investigated at 2 μm, as also described in a previous work [23]. The cumulative amounts of drug which permeated in vitro at 24 h (Q_24_) served as an indicator of the ability of the formulations to deliver the active in vitro. The Q_24_ values (μg/cm^2^) were plotted (i) against the amount of NIA (mg) per amount of protein (g) inside the skin in vivo, and (ii) against the concentration of NIA inside the skin (mg/cm^3^) in vivo (Figure 5).

**Figure 6:**
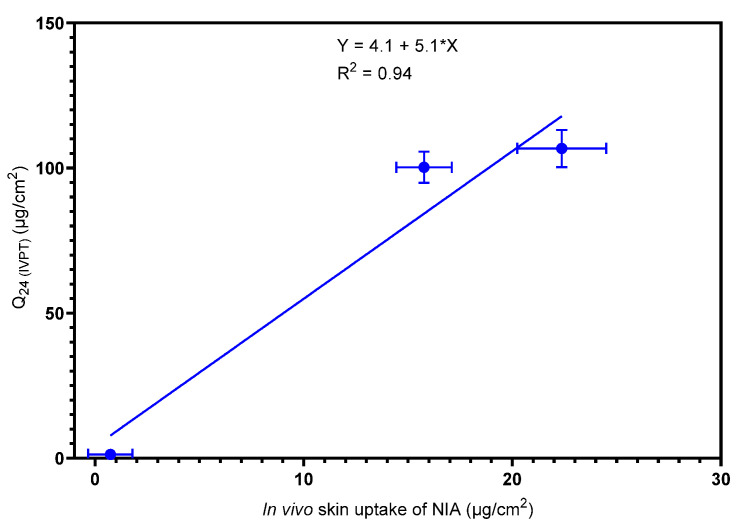
Correlation of the in vitro cumulative amount of NIA permeated after 24 h (4 ≥ *n* ≥ 3; mean ± SEM) and the total amount of NIA in the SC per skin surface area after 60 min in vivo (*n* = 8; mean ± SEM).

A positive correlation (R^2^ = 0.98) was found between the amount of NIA (mg) per skin protein (g) or the NIA concentration inside the skin in vivo (mg/cm^3^) at a depth of 2 μm and the cumulative permeation in vitro. This correlation indicates the considerable potential of CRS for use in skin research; however, the depth selected for investigation can vary between different researchers and different formulations [22,23]. For CRS to be widely used for examining skin delivery, a more robust approach would be needed that enables a common protocol for representing the results and conducting the studies. For this, the total uptake of drug in SC can be calculated as described in a previous report [24]. Here, the total amount of drug (μg) per unit area (cm^2^) after the 60 min application of formulations was plotted against the corresponding in vitro cumulative permeation, and the results are shown in Figure 6.

The total amount of NIA that penetrated the SC in vivo from PG:PGML:IPM was 22.4 μg/cm^2^. This value was significantly higher compared with the NIA permeation from PG:PGML (15.8 μg/cm^2^, *p* < 0.05). Both systems promoted a significantly higher delivery of NIA than neat TC, at 0.7 µg/cm^2^, (*p* < 0.05). The total amount of NIA in the SC per unit of surface area in vivo correlated very well with the cumulative NIA permeation in vitro, with a Pearson correlation coefficient (R^2^) of 0.94.

We previously published a study where the permeation of NIA through human skin in vitro was positively correlated with the in vivo skin uptake using semi-quantitative CRS [22]. Additionally, an IVIV comparison of the percutaneous absorption of salicylic acid was reported by Mateus et al. [23], and a good correlation was also reported when the cumulative permeation of the drug in vitro was plotted against the CRS signal in the skin. However, these researchers used semi-quantitative CRS, and the permeation of drug in vivo was measured in arbitrary units of intensity. Additionally, the in vitro permeation studies were conducted either under infinite dose conditions or in porcine skin. Here, for the first time we used human skin and finite dose conditions for all experiments, and we report an excellent linear correlation between the cumulative permeation in vitro and skin uptake using a quantitative Raman spectroscopy approach in vivo.

As a consequence of the barrier function of the SC, large differences exist in the material concentrations at different depths in the SC. A high spatial resolution and optimized sensitivity of detection enables CRS to precisely monitor these variations as a function of depth, which creates the basis for the accurate quantitative in vivo measurement of skin uptake. An alternative technique has recently been demonstrated using coherent anti-Stokes Raman scattering (CARS) for the in vivo quantitative penetration of deuterated glycerol in the skin [41]. The method required deuterated active ingredients to create a signal that can be detected by CARS. This limits the method to very specific applications, and this approach has also not been validated against conventional methods for the assessment of topical drug delivery to skin.

## 4. Conclusions

This is the first study to assess the capability of a quantitative CRS in vivo technique to be validated by a well-established in vitro method for the assessment of percutaneous absorption. The skin delivery of NIA was examined using 24 h finite dose Franz-diffusion studies in vitro and CRS following 60 min of application in vivo. An excellent correlation (R^2^ = 0.98) was found between the cumulative permeation of NIA in vitro and the skin uptake of NIA at a 2 μm depth in vivo. Similarly, a very good correlation was also found for the total amount of NIA in the SC per skin surface area in vivo and the cumulative permeation of NIA in vitro (R^2^ = 0.94). Clearly, the small sample size indicates that further experimental work is required; however, the findings are encouraging for the establishment of quantitative CRS as a non-invasive method to evaluate skin delivery in vivo. Additionally, the IVIV correlations underline the potential of using quantitative CRS as a novel approach in bioequivalence testing. To further investigate this hypothesis, we intend to conduct a bioavailability study in vivo with more subjects and examine how the CRS results can correlate with these data. Additional studies on IVIV correlations using other drugs and different vehicles are ongoing.
